# Cu_2_O as an emerging semiconductor in photocatalytic and photoelectrocatalytic treatment of water contaminated with organic substances: a review

**DOI:** 10.1039/d0ra06858f

**Published:** 2020-10-05

**Authors:** Babatunde A. Koiki, Omotayo A. Arotiba

**Affiliations:** Department of Chemical Sciences, University of Johannesburg South Africa oarotiba@uj.ac.za; Centre for Nanomaterials Science Research, University of Johannesburg South Africa

## Abstract

A wide range of semiconductor photocatalysts have been used over the years in water treatment to eliminate toxic organic substances from wastewater. The quest for visible or solar light driven photocatalysts with striking merits such as wide range of applications, ease of preparation, tailored architecture that gives rise to improved performance, ability of dual existence as both p type or n type semiconductor, among others, presents copper(i) oxide as a promising photocatalyst. This paper reviews the recent applications of Cu_2_O in photocatalytic and photoelectrocatalytic treatment of water laden with organic pollutants such as dyes and pharmaceuticals. It covers the various modes of synthesis, morphologies and composites or heterostructures of Cu_2_O as found in the literature. Concluding remarks and future perspectives on the application of Cu_2_O are presented.

## Abstract 2

A wide range of semiconductor photocatalysts have been used over the years in water treatment to eliminate toxic organic substances from wastewater.

## Graphical abstract

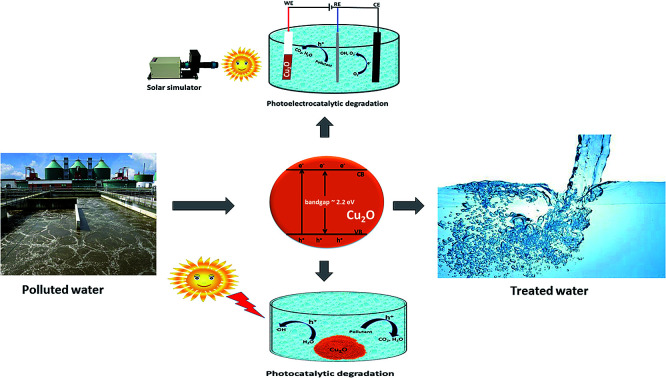

## 1. Introduction

The increase in pollution of water is a global concern owing to its adverse effect on life and on the environment. This increase also adds to the challenges in water treatment owing to the recalcitrant nature of some of these pollutants. Organics such as dyes, pharmaceuticals, endocrine disruptors, surfactants, personal care products *etc.*, constitute a major classification of pollutants and have been known to be difficult to remove from water. For example, organic dyes and their intermediates can undergo various reactions such as oxidation and hydrolysis among others, giving rise to carcinogenic, mutagenic or teratogenic compounds which have damaging effects on microorganisms, aquatic life, soil and water. Various studies have revealed that since the beginning of the second century, there has been a notable increase in the widespread use of pharmaceuticals in the water environment.^1–3^ Wastewaters laden with recalcitrant organic dyes are widely known to possess high chemical oxygen demand (COD), intense color, total dissolved solids (TDS) content, inconsistent pH and low biodegradability.^4^ Globally, pharmaceuticals such as antipyretics, analgesics, blood lipid regulators, antibiotics, antidepressants, chemotherapy agents, and contraceptives are consumed. These drugs (either as free or metabolites) are transported *via* various means to water treatment plants. Studies have shown that owing to complete removal during treatment, these organics eventually find their way into drinking water sources such as surface water and ground water.^5–10^

A clean and safe environment that is free of air, water and soil pollution of no doubt is a necessity for human health and survival. In view of this, the need to develop efficient and environmentally friendly means of treating water laden with toxic organic substances is inevitable. Conventional water treatment techniques have been found to be inefficient owing to sludge formation, generation of secondary pollutants, incomplete removal of pollutants, transfer of pollutants to another medium, *etc.*^11^ On the other hand, advanced oxidation processes (AOPs) have proven to be efficient in the degradation of harmful and highly resistant pollutants due to the *in situ* generation of strong oxidising agent, such as hydroxyl radicals (˙OH), which possesses the ability to completely mineralise toxic organic pollutants. There are four known methods utilized in AOPs to generate ˙OH – ozonation, electrochemical process, direction decomposition of water and photocatalysis.^12–15^

Photocatalyis (PC) seems to distinguish itself as a viable technique in wastewater treatment owing to the ease of use, versatility, non-toxicity, ability to completely mineralise recalcitrant contaminants, environmental friendliness, and the elimination of target pollutants without transfer from one medium to another. Photocatalysis has been employed lately in treating pollutants such as dyes,^16–19^ pharmaceuticals and other endocrine disruptors.^20–25^ Basically, it is a process in which a photocatalyst (semiconductor), when irradiated with photons greater than the bandgap of the semiconductor, gives rise to electron excitation from the valence band (VB) into the conduction band (CB), resulting into photogenerated electrons and holes. The holes being powerful oxidants, can react directly with the toxic organics to break them down, as well as react with water to generate ˙OH which also degrade organics. The electrons on the other hand, react with dissolved oxygen to produce either superoxide or hydroperoxide radicals.^26^

Another subset of AOP is photoelectrocatalysis (PEC), a process that seeks to obtain a synergy in the combination of photocatalysis and electrocatalysis. PEC possesses some advantages over PC, and these include easy reusability of the electrode as compared to the catalyst powder in PC such as the possibility of reduced recombination rate owing to the application of bias potential thus increasing the mineralisation of organics in wastewater. PEC also offers the generation of reactive oxygen species at the cathode and this plays a positive role in the degradation of organics. In a bid to utilize the free energy source, sunlight can be used to drive PEC and this is known as solar PEC.^27^

Cu_2_O has been widely adjudged as one of the promising visible light active semiconductors whose application cuts across areas such as: photocatalysis,^28^ photoelectrocatalysis,^29^ water splitting,^30^ hydrogen evolution,^31^ sensor applications,^32^ solar cells^33^ and Li-ion battery.^34^ Cu_2_O possesses a good solar energy harvesting potential owing to the fact that it has a narrow band gap (2.0–2.4 eV). It is cheap, generally abundant, non-toxic, easy to synthesise, environmentally friendly and absorbs better in the visible light region.^35–38^ Cu_2_O can exist in the n-type nature, having the electrons as the major carriers and holes as the minor carriers if synthesised under acidic condition. It can also exist in the p-type nature, possessing holes as the major carriers and electrons as the minor carriers if prepared under basic condition showing the role that precursors play in the nature of the product obtained.^39^ Reports have shown that Cu_2_O, at room temperature has a high hole-mobility of 50–100 cm^2^ V^−1^ s^−1^,^40–42^ large carrier diffusion length of 2–12 μm,^43^ high carrier concentration of 10^16^–10^19^ cm^−3^,^28,44,45^ and when mixed with other semiconductors with band gap that aligns, it results into the formation of type-II band alignment. These features are required to efficiently improve photogenerated electrons–hole pair separation thus giving rise to increased efficiency in degradation process.^39^

The synthesis, morphologies, and applications of Cu_2_O in photocatalytic and photoelectrocatalytic water treatment methods are discussed in this review.

### 1.1 Cu_2_O: synthesis routes and morphologies

Tailorable architecture is a unique ability possessed by Cu_2_O as evident from the possibilities of different synthesis pathways and resulting morphologies. Over the years, several materials and methods of synthesis of Cu_2_O have been explored by researchers leading to different particle size, and morphology. These methods along with synthesis precursors are catalogued in Table 1.

**Table 1:** Various synthetic routes and morphologies

Method	Materials	Particle size	Morphology	Ref.
Sol gel	Copper acetate, ethylene glycol, NaOH	∼3 nm	Cubic	49
Photochemical reduction	Copper acetate, ethylene glycol, polyethylene glycol	∼500 nm	Cubic	58
Chemical precipitation	Copper sulfate, glucose, NaOH	∼1 μm	Truncated cube	51
One-pot template free	Copper acetate, ascorbic acid, NaOH	20–500 nm	Nanocubes	59
Electrodeposition	Copper sulphate, citric acid, NaOH	∼1 μm	Cubic	29
Spray pyrolysis	Copper nitrate, glucose, 2-propanol	80 nm	Spherical	60
Hydrothermal	Copper acetate, acetic acid, *o*-anisidine	60–100 nm	Nanowire	61
Eco-friendly (green synthesis)	*Aloe vera* leaves, copper sulfate, NaOH	24–61 nm	Mixed truncated	56
			Octahedral	
			Spherical	
Microwave	Copper nitrate, EDTA, NaOH	5–10 μm	Square and spike	62
Sputtering	Copper target, radio frequency power supply	62 nm	Triangular pyramid	63
Wet chemical reduction	Copper acetate, NaOH, ascorbic acid	∼500 nm	Ball-like	64
Solvothermal	Copper acetate, urea, propanetriol, ethanol	1.5–2 μm	Octahedral	65
Low temperature treatment	Copper acetate, sodium tartrate, glucose, NaOH	1.5–2 μm	Octahedral	66

#### 1.1.1 Hydrothermal

Among the well-known methods used for the synthesis of Cu_2_O, hydrothermal route appears to stand out owing to the fact that it gives rise to well crystalline form of Cu_2_O. In addition, by simply optimising the reaction pressure, temperature, time and solution pH, the morphology of the Cu_2_O obtained can be controlled. While Pan *et al.*^46^ prepared Cu_2_O film from CuCl_2_, Cu(NO_3_)_2_ and CuSO_4_ hydrothermally with copper plate to obtain rod like Cu_2_O, Dong *et al.*^47^ on the other hand simply synthesised Cu_2_O from metallic copper in the presence of graphene oxide as an oxidant. As much as the product formed by Dong *et al.* possessed improved stability, the different graphene oxide composition will likely pose an effect on how well this method can be reproduced. The search for a hydrothermal route that is free of additional oxidising agent led Zimbovskii and coworkers to propose a better pathway in which the oxygen that is directly present in the solution as well as the gas phase of the hydrothermal cell would act as an oxidant. In simple terms, metallic copper was hydrothermally treated in the presence of 0.3 M NaOH solution at 180 °C for 1 h. The resulting Cu_2_O formed was polyhedral in shape with average particle size of 2 μm.^48^

#### 1.1.2 Sol–gel

**Fig. 1:**
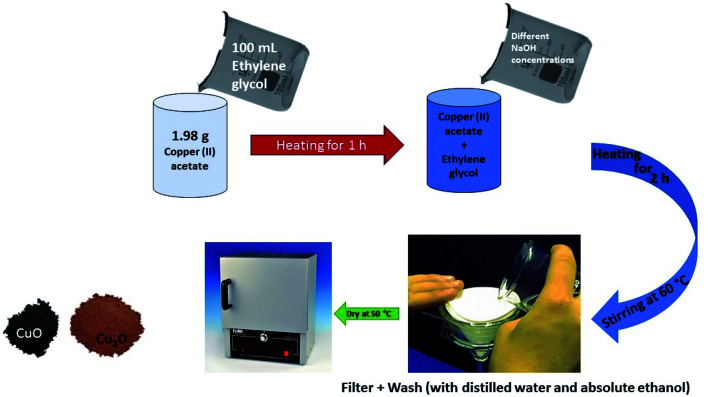
Scheme showing the synthesis of Cu_2_O and CuO by sol–gel method.

Copper is known to be more stable in its +2 state than +1 oxidation state. It is therefore difficult to obtain a pure form of Cu_2_O without the presence of impurities. In the quest to obtain pure form of Cu_2_O, quite a number of physical and chemical methods have been used, but they entail high temperatures, extended reaction time, state-of-the-art equipment, inert atmosphere or reducing agents. The need to obtain a stable and pure form of Cu_2_O in the absence of additives or surfactant led Zayyoun and co-workers to explore the possibility of a sol–gel synthesis approach.^49^ The schematic representation of the synthesis is illustrated in Fig. 1. It was reported that at pH lower than 7, cubes representing pure Cu_2_O were formed. However, on increasing the pH towards the basic medium, black spherical CuO structures were formed. The XRD confirmed the absence of impurities in the product formed and the UV-vis spectra showed that the product formed showed interesting optical properties, thus suggesting its stability in harvesting solar energy.^49^

#### 1.1.3 Electrodeposition

Electrodeposition method provides a simple, safe, low cost route of obtaining high purity Cu_2_O. Zheng *et al.*^50^ obtained an octahedron shaped Cu_2_O by electrodepositing Cu_2_O on a substrate in a three-electrode system with 0.02 M copper acetate and 0.08 M sodium acetate serving as electrolyte solution. The electrodeposition was carried out at −0.1 V and solution pH of 5.0 ± 0.1 between 10 and 50 min reaction time. It was conclusively gathered that the reaction time of 30 min gave rise to a closely packed Cu_2_O octahedrons. On the other hand, Koiki *et al.*^29^ obtained cubic shaped Cu_2_O using CuSO_4_·5H_2_O and citric acid at −1 V. The electrolyte solution pH was maintained at 11 for 10 min reaction time. Comparing the two routes, we can infer that the precursor, solution pH and deposition potential played a significant role in the morphology of the product obtained.

#### 1.1.4 Chemical reduction

**Fig. 2:**
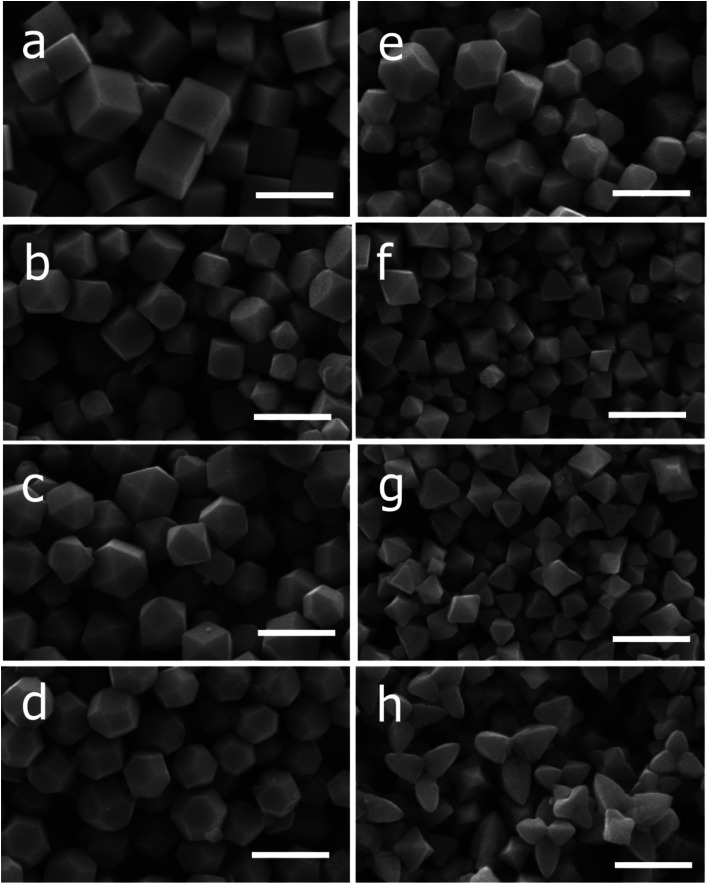
SEM images of the Cu_2_O nanocrystals with various morphologies with vol. of NH_2_OH·HCl added (in parenthesis): (a) cubes (0.15 mL), (b) truncated cubes (0.25 mL), (c) cuboctahedra (0.35 mL), (d) type I truncated octahedra (0.45 mL), (e) type II truncated octahedra (0.55 mL), (f) octahedra (0.65 mL), (g) short hexapods (0.95 mL), and (h) extended hexapods (0.76 mL). Scale bar = 1 μm (this figure has been adapted/reproduced from ref. 53 with permission from AMERICAN CHEMICAL SOCIETY, copyright 2009).

The report of Zhang *et al.* showed that the choice of synthesis route of Cu_2_O can play a vital role in the morphology and also in the rate of degradation.^51^ It was reported that a cubic shaped Cu_2_O obtained by photochemical reduction method displayed 70% degradation rate. Further studies showed that spherical, irregular lumped, octahedral, cubic and truncated cubic shaped Cu_2_O all obtained by chemical precipitation method gave rise to 50%, 37%, 100%, 55% and 57% pollutant removal respectively.^51^ Worthy of note is the fact that the synthesis time also influences the morphology of Cu_2_O. Kuo and Huang^52^ reported a facile, cheap aqueous colloidal solution route in the synthesis of Cu_2_O in the presence of a reductant to achieve different morphologies for Cu_2_O nanocrystals. The sequence of synthesis was: water, CuCl_2_, sodium dodecyl sulfate (SDS), NH_2_OH·HCl and NaOH; where NH_2_OH·HCl served as a reductant. The resultant solution was aged for 2 h to obtain the desired product. The process involved in the formation of Cu_2_O nanocrystals as well as the resulting morphologies was studied by a close examination of the intermediate products. Particles showing resemblances of a cube, cuboctahedron, and octahedron were formed after aging for 5 min. Although they possessed rough surfaces, it was still indicative that an instantaneous growth had taken place. It was assumed that by varying the amount of the NH_2_OH·HCl, the growth rate towards the (100) direction with respect to (111) direction may have been influenced. Due to ripening and surface reconstruction that took place, the final products were formed possessing unique shapes.^52^ Progressively, Ho and Huang^53^ reported the synthesis of cubic, octahedral and hexapod morphologies by changing the sequence of precursors as follows: water, CuCl_2_ solution, NaOH, SDS and NH_2_OH·HCl. The reaction pH and volume were kept at 7 and 10 mL respectively, while varying the volume of the 0.2 M NH_2_OH·HCl reductant added. This was done to allow the Cu(OH)_2_ and Cu(OH)_4_^2−^ species present in the solution to thoroughly mix before the addition of the reductant. The resulting morphologies with the volume of reductant added in this synthesis is presented in Fig. 2.

#### 1.1.5 Green synthesis

Synthesis of Cu_2_O based on green approach is increasingly drawing the attention of researchers. Green synthesis of Cu_2_O primarily entails the use of extracts from plants. Its merit hinges on the fact that the plant extract used is readily available, non-toxic when handled and serves a dual function of both reductant and stabiliser. In addition, it has a high product yield. Ramesh and coworkers^54^ obtained a spherical and semi spherical Cu_2_O nanoparticles using leaf extract of *Arachis hypogea* L, Abboud *et al.*^55^ also obtained a spherical Cu_2_O nanoparticles but with *Bifurcaria bifurcate*, a marine alga. Kerour *et al.*^56^ obtained truncated octahedral, octahedral and spherical like Cu_2_O nanoparticles by simply varying the concentration of the extract used. Basically, 0.25 g mL^−1^, 1.5 g mL^−1^ and 3.5 g mL^−1^ of *Aloe vera* leaf plant extracts were contacted CuSO_4_·5H_2_O, followed by 40 mL of 2 M NaOH solution. The mixture was continuously stirred for 25 min at 130 °C to obtain a brick red precipitate of Cu_2_O.^56^ It can therefore be gathered that plant extract concentration plays a significant effect on the morphology of Cu_2_O nanoparticles.

#### 1.1.6 Facile template free

Despite the various successful approach by many researchers in synthesising Cu_2_O, quite a number of these routes entail the use of catalyst or surfactants. The major setback posed by this can be seen in the laborious washing and product harvesting process involved after synthesis. The presence of impurities that can possibly hamper Cu_2_O performance is occasionally unavoidable. Han *et al.*^57^ were able to arrive at a simple, rapid, one-pot and template free synthesis approach for the fabrication of Cu_2_O truncated octahedra. From the synthetic route, a consistent crystalline Cu_2_O truncated octahedra framed by 8 hexagonal (111) and 6 square (100) surfaces was synthesised. Cu_2_O truncated octahedron was formed within 10 min reaction time, but as the reaction time increased, the (100) surfaces became etched with bigger holes giving rise to pits.^57^

### 1.2 Cu_2_O and its facet effects

**Fig. 3:**
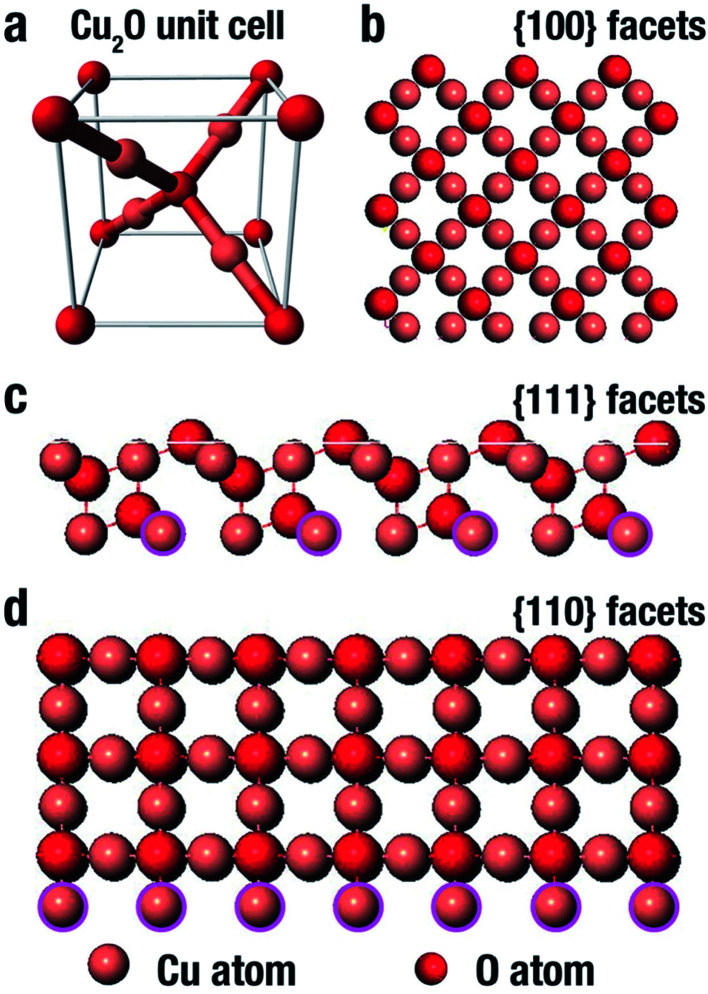
Representation of the (a) Cu_2_O unit cell, and (b–d) Cu_2_O crystal structure (100), (111) and (110) facet respectively.^74^

Cu_2_O comes in a cubic crystal structure with each oxygen atom surrounded by four copper atoms, and each of these copper atoms are bonded by two oxygen atoms as shown in the unit cell (Fig. 3a).^67^ It has been well proven that for the three basic facets of Cu_2_O; (100), (111) and (110), the surface energy is a function of the density of the copper atoms at the edge.^68^ The (100) facet illustrated in Fig. 3b shows that only oxygen atoms are present at the edge, thus indicating that it is electrically neutral.^69^ On the other hand, the (111) facet is positively charged due to the fact that each two copper atoms possess a dangling bond which is at 90° as shown by the pink circles in Fig. 3c, thereby making it easy to interact with negatively charged molecules.^70^ In the same manner, according to Fig. 3d, the (110) facet has the same copper atom being terminated by a dangling copper atom as shown by the pink circles. Comparing the (110) facet plane with (111) facet, it can be seen that the number of dangling copper atoms per unit surface area in (110) is around 1.5 times more than the number of dangling copper atoms per unit surface area on the (111) facet plane.^68^ This therefore suggests that the (110) facet is more positively charged than the (111) facet. Therefore the surface energies for the three facets of copper are in this order: *γ*_(100)_ < *γ*_(111)_ < *γ*_(110)_.

Worthy of note also is the fact that the photocatalytic efficiency of Cu_2_O is dependent on its facet structure. As reported by Ho and co-workers,^53^ a negatively charged molecule, methyl orange, was used to investigate the photocatalytic performance of the different facet of Cu_2_O crystals. It was gathered that the as synthesised octahedral Cu_2_O crystal with purely (111) facet was more photocatalytically active than truncated cubic crystals that had majorly (100) facet. Their findings suggest that Cu_2_O octahedra, which are positively charged will strongly interact with negatively charged molecules, thereby enhancing the photodegradation performance. While cubic Cu_2_O which is electrically neutral did not interact with the molecules present in the aqueous medium, and as such displayed no photodegradation ability. On the other hand, when a positively charged molecule, such as methylene blue was used, both the octahedra and cubes did not show any photodegradation ability.^53^ There was however a notable discovery in their work as they observed that the photocatalytic efficiency of the extended hexapods structure was remarkably high due to the pronounced sharp edges between the (111) facets. A combination of both experimental and computational study can also be used to investigate the turning architecture as well as facet orientation of Cu_2_O nanoparticles.^71,72^ While surfactant such as SDS can be used to tune the growth and morphology of Cu_2_O due to its selective adsorption on different facet density functional theory (DFT) has been generally used to investigate the surface energy of the different orientations, terminations and reconstructions. Su *et al.*^73^ used hydroxylamine hydrochloride and SDS to control the architecture and exposed faces of Cu_2_O nanoparticles. In their study, cubic 26-facet and truncated rhombic dodecahedral structures were formed as the concentration of hydroxylamine hydrochloride was increased in the absence of SDS. However, in the presence of SDS, a development was experienced in the morphology as the 26-facet became porous octahedron and the truncated rhombic dodecahedral transformed into rhombic dodecahedron thus suggesting the selective adsorption of SDS. This further showed that preferential adsorption of SDS onto the surface of three facets of Cu_2_O reduced the surface free energy of copper terminated surfaces giving rise to the active facets that has a high-density Cu dangling bonds and ultimately enhancing the photocatalytic ability. It is therefore crucial to comprehend the tunable architecture of Cu_2_O with respect to its facet as this will help in the synthesis of highly efficient Cu_2_O nanoparticles for photocatalytic applications.^73^

### 1.3 Cu_2_O: composites and heterojunctions

Copper(i) oxide, despite its unique properties, suffers a major setback: owing to its narrow band gap, the photogenerated electrons could rapidly recombine with the holes thereby resulting into low quantum efficiency. The formation of composites, where some are heterostructures, have been devised as some of the possible ways of enhancing the photocatalytic performance of Cu_2_O (Table 2). The hybridization of Cu_2_O with metals, non-metals, metal oxides, carbon-based materials and plasmonic nano-metals such as gold and silver to form heterostructured composites has been extensively explored. Formation of composites presents the possibility of promoting charge carrier mobility, leading to the generation of an internal electric field, thereby improving the separation of the charge carriers culminating into improved Cu_2_O performance as a photocatalyst. In addition, in a Cu_2_O composite, the dopant could serve as an electron sink. The photo-generated electrons of Cu_2_O could be channeled into the dopant resulting into reduced recombination rate. This in turn gives rise to production of more oxidants (h^+^ and OH) for degradation the toxic organics. This approach, despite its merits, suffers setback as the dopants may in turn bring about negative effect arising from new recombination centers caused by new deep introduced into the bandgap.^75^ The formation of composites containing heterojunctions on the other hand, provides a more efficient way of separating the photogenerated charge carriers. Basically, heterojunction is formed between two semiconductors with different band structures, resulting into band alignment. This approach improves the photocatalytic activity of the photocatalyst through improved light harvesting, enhanced charge separation, and charge carries lifetime extension. A review discussing the semiconductor heterojunction extensively has been published by our group.^76^

**Table 2:** Modification approach and materials^a^

Modification approach	Materials	References
**Composite**		
• Metal/Cu_2_O	Cu@Cu_2_O	77
• Non-metal/Cu_2_O	N–Cu_2_O	78
• Metal oxide/Cu_2_O	CuO/Cu_2_O	79
• Hybrid/Cu_2_O	Carboxymethyl cellulose/Cu_2_O	80
• Plasmonic nano-metal/Cu_2_O	Au/Cu_2_O and Ag/Cu_2_O	81 and 82
• Carbon NM/Cu_2_O	Cu_2_O–RGO	51

**Heterojunctions**		
• Type II p–n	g-C_3_N_4_/Cu_2_O	28
• p–n–p	BiOCl/g-C_3_N_4_/Cu_2_O/Fe_3_O_4_	83

a NM = nanomaterials such as nanotubes, quantum dots and graphene.

## 2. Cu_2_O and its catalytic applications in water treatment

Most Cu_2_O applications till date have been in the area of CO_2_ reduction, water splitting, energy conversion and fuel cell, among others. While its application in the photocatalytic and photoelectrocatalytic degradation of dyes and most especially pharmaceuticals can be said to be fewer. These water treatment applications are reviewed in the following sections.

### 2.1 Cu_2_O: photocatalytic degradation

In recent times, toxic organic pollutants such as dyes, pharmaceutical and personal care products have drawn the attention of researchers owing to their accumulation in the environment and their devastating effect to human health. The photocatalytic application of Cu_2_O for pollutant degradation is dated back to 2005 when Yu *et al.* synthesised and applied Cu_2_O nano-whiskers for the degradation of *p*-chloronitrobenzene.^84^ This later paved the way for further works to be carried out in the area of PC and PEC. Cu_2_O has been explored in the remediation of water polluted with toxic organic pollutants such as dyes and pharmaceuticals as summarised in Table 3.

**Table 3:** Recent studies on photocatalytic and photoelectrocatalytic degradation toxic organics involving Cu_2_O^a^

Materials	Method of preparation	Analyte	% removal	Rate constant	Ref.
**Photocatalysis**					
Cu_2_O and Cu_2_S	Co-precipitation and calcination	Congo red (200 mg L^−1^)	99.8%	NR	85
		Methyl orange (50 mg L^−1^)	90.1%		
		Tetracycline (50 mg L^−1^)	84.8%		
Core@shell Ag_3_PO_4_@Cu_2_O	Liquid phase reduction and chemical deposition	Methylene blue (20 mg L^−1^)	97% after 20 min	NR	94
KAPs-B/Cu_2_O	Precipitation	Methyl orange (30 mg L^−1^)	∼92% within 60 min	NR	95
Cu_2_O/Ag/AgCl	Oxidation	Methyl orange	93% within 16 min	NR	96
Cu_2_O/TiO_2_	Electrodeposition	Rhodamine B (30 mg L^−1^)	98.4% after 180 min	0.0230 min^−1^	97
BiOCl/g-C_3_N_4_/Cu_2_O/Fe_3_O_4_	Co-precipitation	Sulfamethoxazole (100 μM)	99.5% within 60 min	0.0543 min^−1^	83
Cu_2_O/PSF membrane	Electrodeposition	Ibuprofen	86% within 60 min	0.03263 min^−1^	98
Cu_2_O hallow nanospheres	Hydrothermal	Methylene blue (100 mg L^−1^)	∼92% within 10 min	NR	99
Fe_3_O_4_/SiO_2_/Cu_2_O–Ag	Ultrasound-assisted precipitation	Rhodamine B (3 × 10^−5^ M)	94.35% after 90 min	NR	100
g-C_3_N_4_/Cu_2_O	Sol–gel	Methylene blue (1 × 10^−5^ M)	81% after 120 min	0.0112 min^−1^	101
		Rhodamine B (1 × 10^−5^ M)	85.3% after 120 min	0.0125 min^−1^	
CuO–Cu_2_O/GO	Hydrothermal	Tetracycline (10 mg L^−1^)	90% after 120 min	0.0205 min^−1^	51
		Methyl orange (10 mg L^−1^)	95% after 120 min	NR	

**Photoelectrocatalysis (analyte and experimental condition)**					
Cu_2_O/α-Fe_2_O_3_	Electrodeposition	Oxytetracycline (10 mg L^−1^). 0.5 V bias potential using xenon lamp	73.3% after 60 min	0.0214 min^−1^	89
Cu_2_O/TiO_2_	Electrodeposition	2,4,6-Trichlorophenol (5 mg L^−1^). 1.0 V bias potential and 35 W xenon lamp	99.9% within 120 min	NR	102
Cu_2_O/TiO_2_	Chemical bath deposition	Ibuprofen (10 mg L^−1^). 1.0 V bias potential and 100 W Hg lamp	100% after 120 min	0.0464 min^−1^	90
Cu_2_O/TiO_2_	Electrochemical anodisation and pulse electrodeposition	Chloramphenicol (10 mg L^−1^). 0.5 V and 300 W xenon lamp	66.8% removal within 240 min	0.00875 ± 0.00049 min^−1^	103
Cu_2_O/TiO_2_	Ultrasound-assisted successive ionic layer adsorption and reaction (SILAR)	Methyl orange	77.62%	0.0086 min^−1^	104
		Rhodamine B	61.83%	0.0053 min^−1^	
		Methylene blue within using 500 W xenon lamp	98.30%	0.037 min^−1^	
Cu_2_O/TiO_2_ NTA	Electrodeposition	Ciprofloxacin (10 mg L^−1^). 1.5 V bias potential	73% removal after 240 min	0.00605 min^−1^	29
n-ZnO/p-Cu_2_O/n-TNA	Electrodeposition	Tetracycline (20 mg L^−1^). 0.5 V bias potential using a xenon lamp	90% removal after 180 min	NR	92
Cu_2_O/Au/TiO_2_ NAs	Electrodeposition	Methyl orange (10 mg L^−1^) using 300 W xenon lamp	90% after 240 min	NR	105

a NR = not reported.

The formation of heterojunction improves the photocatalytic activity of the photocatalyst due to the internal electric field created at the interface of the semiconductors. Owing to the solar light harvesting potential and better visible light absorption of Cu_2_O, Zuo *et al.* synthesised Cu_2_O/g-C_3_N_4_ p–n heterojunctioned photocatalyst for the degradation of methyl orange. Their findings showed that the heterojunction photocatalyst possessed larger surface area which gave rise to multiple active sites for photocatalytic reaction. A removal efficiency of 84% was obtained within 30 min and the photocatalyst was found to be stable over five runs.^37^

Cu_2_O possesses an excellent adsorptive and photocatalytic ability in the removal of non-biodegradable organics in wastewater. The preparation, adsorptive and photocatalytic application of Cu_2_O and its nanocomposite have been studied by Yue *et al.*^85^ Different mass ratios of Cu_2_O/Cu_2_S photocatalyst were used to degrade the toxic organic pollutants under visible-light irradiation. The Cu_2_O/Cu_2_S-9/1 gave the best performance after degrading 99.8% solution containing 200 ppm Congo red, 90.1% solution containing 50 ppm methyl orange and 84.8% solution containing 50 ppm tetracycline under 120 min. It was gathered that this nanocomposite displayed a more excellent degradation performance when compared to others works that have been previously reported. This therefore suggests Cu_2_O/Cu_2_S nanocomposite as a promising photocatalyst for the removal of organic pollutants. Furthermore, its reusability was examined over four consecutive cycles and the percentage removal was still constant around 90%, thus suggesting the as-synthesised nanocomposite as a stable visible light driven photocatalyst.^85^

While Yue *et al.* explored the adsorptive and photocatalytic ability of Cu_2_O/Cu_2_S nanocomposite, Koiki *et al.* in their work replaced the Cu_2_S with g-C_3_N_4_ to from Cu_2_O/g-C_3_N_4_ p–n heterojunction for the photocatalytic degradation of orange II dye in water. The synthetic route was novel and the reaction time was shorter. The Cu_2_O/g-C_3_N_4_-9:1 displayed the best photocatalytic performance resulting to a removal efficiency of 85% which was about 3.6 times higher than pure Cu_2_O. Its mineralisation efficiency was around 60% and the photocatalyst was found to be stable.^28^

Among the various graphene-related materials, exfoliated graphite (EG) is a choice support and electromagnetic shielding material for photocatalysts.^86^ The formation of heterostructure using exfoliated graphite as support material for Cu_2_O for the photocatalytic degradation of methyl orange was reported by Zhao *et al.* It was gathered that the EG availed a 3D environment for photocatalytic reaction and conferred a high adsorption capacity for Cu_2_O. The removal efficiency of about 96.7% was obtained for Cu_2_O/EG (10 wt%). The photocatalyst was found to be stable after five cycles.^87^

Heterostructure formation involving a metal and Cu_2_O is an efficient approach to reduce recombination of photogenerated charge carriers and to improve the photocatalytic efficiency of Cu_2_O. Sun *et al.* used Ag/Cu_2_O for the degradation of methylene blue. The tailored heterostructure synthesised by the etched Cu_2_O truncated octahedrons showed improved photocatalytic activity that was 3.1 and 5.7 times better than etched Cu_2_O truncated octahedrons and pristine Cu_2_O truncated octahedrons. This enhanced performance can be attributed to the electron sink effect of silver nanoparticle.^88^

In other works, the formation of a quaternary magnetic nano-junction involving Cu_2_O, BiOCl, g-C_3_N_4_ and Fe_3_O_4_ was used to study the effects of visible light and solar light in the degradation of pharmaceuticals from wastewater. Kumar *et al.*,^83^ explored the possibility of degrading sulfamethoxazole (SMX) by utilizing light from the sun and visible light as both natural and artificial light source. It was reported that under visible light, 99.5% of the SMX was degraded within 60 min, while under natural sunlight, 92.1% of the SMX was degraded under 120 min. It is quite noticeable that there was a reduction in the rate of degradation with a corresponding time extension on switching form artificial light to natural light. This can be attributed to the fact that the natural light consists of UV, IR, and visible light giving rise, thus making the reaction slower as compared to the artificial light source which contains only visible light. Furthermore, the inconsistent solar intensity during the day could impede the degradation process. Nevertheless, the use of natural light connotes lower cost and sustainability. Scavenger studies showed the hydroxyl radicals as the main active species in the degradation process. The photocatalyst can be said to be relatively reusable having maintained a fair drop in the percentage degradation from 99.5 to 97.0% over five cycles.^83^

### 2.2 Cu_2_O: photoelectrocatalytic degradation

**Fig. 4:**
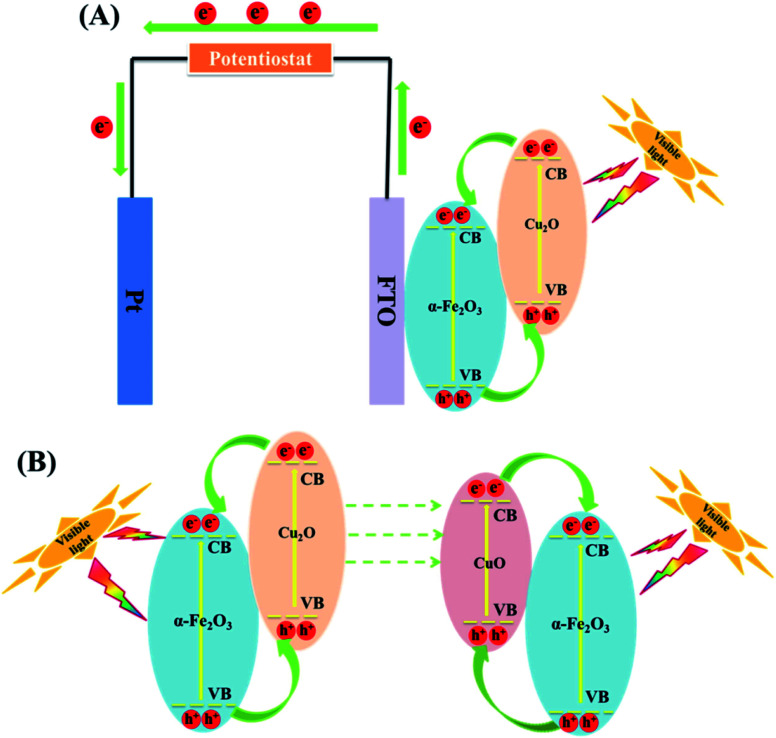
(A) Schematic diagram of photo-induced electron–hole pairs separated process in PEC system based on Cu_2_O/α-Fe_2_O_3_. (B) A mechanism for the changes of Cu_2_O/α-Fe_2_O_3_ after PEC treatment (this figure has been adapted/reproduced from ref. 89 with permission from ELSEVIER, copyright 2018).

In PC, the recovery of the catalyst is a challenge. However, in PEC, the catalysts have been confined unto a substrate and used as an electrode thus mitigating the challenge of catalyst recovery. The PEC application also carries the advantage of the use of electrical potential to further separate the electron. In the preparation of anodes, conductive substrates upon which the Cu_2_O are deposited are needed. Substrates such as titanium sheet, anodised TiO_2_, fluorine doped tin oxide (FTO) have been used. Zhang and co-workers^89^ efficiently degraded oxytetracycline using Cu_2_O deposited on α-Fe_2_O_3_ and fluorine doped tin oxide (FTO) as the conducting substrate. A 10 mg L^−1^ oxytetracycline solution prepared in 0.1 Na_2_SO_4_ solution was used as the electrolyte with a xenon lamp as light source. As the deposition time was varied from 1 to 5 min, the percentage removal also increased from 37.5 to 73.3% within 60 min. This therefore confirmed that deposition time can have effect on the PEC performance of Cu_2_O. The PEC performance of Cu_2_O, Fe_2_O_3_ and Cu_2_O/α-Fe_2_O_3_ were also studied by immobilising them on FTO glass. The composite photoanode displayed the best performance of 73.3% oxytetracycline removal when compared to pure Cu_2_O (26.9%) and α-Fe_2_O_3_ (19.2%) within 60 min. The improved performance of the composite photoanode can be due to the enhanced visible light absorption range and improved charge carrier separation arising from the formation of a Cu_2_O/α-Fe_2_O_3_ p–n heterojunction as shown in Fig. 4. Finally, the electrochemical (EC), photocatalytic (PC) and PEC processes for the abatement of oxytetracycline was studied using the Cu_2_O/α-Fe_2_O_3_ composite photoanode with 60 min. The resulted obtained showed that removal percentages were 20.7%, 39.0% and 73.3% for PC, EC and PEC respectively. The composite photoanode was found to be stable and reusable having shown 15% loss within 5 cycles.^89^ This study shows that PEC can be an improved version of PC in degradation performance.

The need to eliminate recalcitrant pharmaceuticals such as ibuprofen using a highly efficient technique became the drive for Sun *et al.*^90^ where Cu_2_O-doped TiO_2_ nanotube arrays for the photoelectrochemical degradation of ibuprofen was investigated. The Cu_2_O-doped TiO_2_ nanotube arrays showed a better performance by completely degrading the ibuprofen in 120 min as compared to the pure TiO_2_ nanotube arrays showing complete ibuprofen degradation after 240 min. It therefore shows that anchoring the Cu_2_O on the TiO_2_ nanotube array remarkably improved the photogenerated charge carrier separation, thus giving rise to the formation of more holes for the oxidation of ibuprofen in the anodic chamber. While comparing the PEC, EC and PC processes, it was observed that PEC showed a complete ibuprofen removal, while PC and EC showed 53.3% and 11.5% respectively within 120 min. This further strengthens the claim that PEC is more efficient method compared to EC and PC owing to the synergistic effects of EC and PC taking place in PEC.^90^

Photoelectrocatalytic performance of photoanodes can be enhanced by coupling two or more semiconductors into a heterojunction formation. One of the early works that involves the use of Cu_2_O in a heterojunction formation for water treatment was reported by Zewge *et al.*^91^ In this work, Cu_2_O was used to improve the photoelectrocatalytic performance of TiO_2_ in the decolourisation of methyl orange. It was reported that the TiO_2_/Cu_2_O photoanode gave rise to highly efficient photogenerated charge separation, thus improving the PEC degradation. A complete decolourisation was achieved using 1.0 V bias potential in 24 h. The authors concluded that Cu_2_O played a significant role in the degradation of the dye by enhancing the separation of the photogenerated charge carriers and driving the TiO_2_ towards the visible light region for improved light absorption.^91^ While this work centered primarily on TiO_2_, the conclusion actually provided a useful information about the photoelectrocatalytic potential of Cu_2_O.

**Fig. 5:**
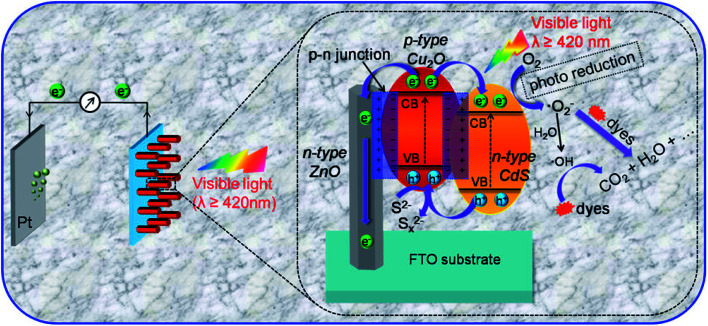
Schematic diagram of the PEC degradation process on n-CdS/p-Cu_2_O/n-ZnO NRAs and a magnified view of the proposed working mechanism (this figure has been adapted/reproduced from ref. 93 with permission from AMERICAN CHEMICAL SOCIETY, copyright 2017).

The literature now has other reports on the use of Cu_2_O in the formation of heterojunctions for PEC application for water treatment (Table 3). For example, Li *et al.*^64^ were among the first to prepare a ternary heterojunction involving n-ZnO/p-Cu_2_O/n-TiO_2_ nanotube array (TNA) for the degradation of tetracycline. The heterojunction was formed by sonoelectrochemically depositing Cu_2_O on the TNA surface. The ZnO was then anchored on the p-Cu_2_O/n-TNA *via* hydrothermal synthesis. The PEC degradation was carried out using both visible light and solar light. Under visible light, the percentage degradation for tetracycline was found to be 75% for p-Cu_2_O/n-TiO_2_ photoanode and 85% for n-ZnO/p-Cu_2_O/n-TiO_2_ ternary electrode within 180 min. However, under solar light, there was enhanced PEC performance, as the percentage removal of tetracycline using the ternary electrode increased to 90% after 180 min. This therefore showed the ternary n-ZnO/p-Cu_2_O/n-TiO_2_ photoanode to be efficient under both visible and solar light sources. We can safely say that the formation of a ternary heterojunction of n-ZnO/p-Cu_2_O/n-TiO_2_ provides enhanced photocatalytic efficiency during PEC process due to the photogenerated charge carrier separation and the reduced rate of recombination with the electrodes. It further lends protective hand to Cu_2_O by preventing it from photocorrosion. Their findings overall suggest n-ZnO/p-Cu_2_O/n-TiO_2_ as a highly efficient, stable, reliable and reusable photoanode in the PEC degradation of pharmaceuticals.^92^ Another possible configuration in photoanode development where a dual p–n junction involving the use of Cu_2_O/ZnO and Cu_2_O/CdS for photoelectrocatalytic degradation is depicted in Fig. 5.

A visible light driven p–n heterojunction photoanode fabricated by electrodepositing Cu_2_O on anodised TiO_2_ nanotube arrays (NTAs) was used by Koiki *et al.*, to degrade ciprofloxacin. The work reported a robust approach to fabricate a highly-ordered TiO_2_ nanotube array (NTA) from titanium sheet. The Cu_2_O films electrodeposited on the TiO_2_ shifted the absorption edge of the TiO_2_ NTA from the UV region to the visible light region. In addition, the formation of the p–n heterojunction created efficient photogenerated charge carrier separation thereby giving rise to a reduction in the recombination rate and an improved pollutant percentage degradation. The as-prepared pure TiO_2_ NTA electrode resulted to 65% ciprofloxacin degradation, while the TiO_2_ NTA/Cu_2_O showed 73% ciprofloxacin removal within 240 min. It can be inferred that composite photoanode gave a better performance due to the introduction of Cu_2_O. The lower bandgap of Cu_2_O and its photocatalytic activity in the visible light region must have contributed to the enhanced light harvesting of the TiO_2_ NTA/Cu_2_O. The composite photoanode was found to be suitable for treating effluents ranging from the slightly acidic to the strongly basic region. Scavenger studies revealed the holes as the major oxidant while conclusively, the photoanode was found to be stable and reusable after 10 runs.^29^ The extent of degradation when Cu_2_O is coupled with TiO_2_ seems to be influenced by the structural morphology of the TiO_2_, with nanotube arrays yielding better performance.^29,91,92^ Other reports on the photoelectrocatalytic application of Cu_2_O in the removal of organic pollutants can be found in Table 3.

## 3. Conclusions and future perspective

The application of Cu_2_O in water treatment based on photocatalytic and photoelectrocatalytic methods have been demonstrated in the literature. It is indeed a material that worths investigating further owing to its light harvesting capability in the visible light region. Thus, with Cu_2_O, PC and PEC researches are advanced towards sustainability. Cu_2_O can exist as p or n semiconductor and the structure (shape and size) varies and/or are easily amenable. These two points suggest that: (a) there are potentials for a variety of applications where morphology and performances can be correlated. (b) The ‘most’ favourable crystal shapes for PC and PEC applications are yet to be ascertained. (c) The material lends itself to various synthesis methods and thus one will expect more novel synthesis routes in the near future. (d) The p–n variability can be exploited in preparing a myriad of heterojunctions with other semiconductors. (e) The p–n variability opens the possibility of using Cu_2_O as both photoanode and photocathode.

While the variation in shapes and crystal structure (facet effects on the photocatalytic performance of Cu_2_O have been reported) may open up more investigations, it can also pose the challenge of comparison especially if crystal shape changes during applications are possible. Thus, the crystal shape or structural integrity of Cu_2_O may be investigated before and after degradation cycles.

In the area of photocatalysis and photoelectrocatalysis, future work should be geared towards the degradation of other class of pollutants other than dye. This review shows that the current applications of Cu_2_O in PEC treatment of organic pollutants in water is still at its infancy. The few reports highlighted here indeed compares well with results from other PEC applications with other types of semiconductors. Thus, more work with Cu_2_O as photoelectrocatalyst is envisaged. It will also be interesting to see fundamental studies on the degradation pattern and pathways of certain pollutants. These studies can shed more light on the preferred structural morphology with time.

## Conflicts of interest

There are no conflicts of interest to declare.

## Supplementary Material
